# Hedgehog signaling promotes sorafenib resistance in hepatocellular carcinoma patient-derived organoids

**DOI:** 10.1186/s13046-020-1523-2

**Published:** 2020-01-28

**Authors:** Siqi Wang, Yang Wang, Xiaodong Xun, Changkun Zhang, Xiao Xiang, Qian Cheng, Shihua Hu, Zhao Li, Jiye Zhu

**Affiliations:** 1 Department of Hepatobiliary Surgery, Peking University People’s Hospital, Beijing, China; 2 Beijing Key Laboratory of HCC and Liver Cirrhosis, Beijing, China; 3 Peking University Center of Liver Cancer Diagnosis and Treatment, Beijing, China; 4 Peking University Institute of Organ Transplantation, Beijing, China

**Keywords:** Patient-derived organoids (PDOs), CD44, Hedgehog signaling, Hepatocellular carcinoma

## Abstract

**Background:**

The mechanism underlying sorafenib resistance in hepatocellular carcinoma (HCC) remains unclear. Accumulating evidence suggests that tumor-initiating cells (TICs) are a pivotal driving force. Both CD44 and Hedgehog signaling play crucial roles in TIC properties in HCC. In this study, we explored the roles of CD44 and Hedgehog signaling in sorafenib resistance and evaluated the therapeutic effect of cotreatment with sorafenib and Hedgehog signaling inhibitors in HCC patient-derived organoid (PDO) models to improve treatment efficacy.

**Methods:**

We collected HCC specimens to establish PDO models. Cell viability and malignant transformation properties were investigated after treatment with different TIC-related inhibitors alone or in combination with sorafenib to evaluate the therapeutic effect in PDOs and cell lines by in vitro and in vivo experiments. Expression levels of Hedgehog signaling proteins and CD44 were monitored to reveal potential relationships.

**Results:**

We demonstrated that our HCC PDO models strongly maintained the histological features of the corresponding tumors and responded to drug treatment. Furthermore, CD44-positive HCC PDOs were obviously resistant to sorafenib, and sorafenib increased CD44 levels. A drug screen showed that compared with Notch, Hippo and Wnt signaling inhibitors, a Hedgehog signaling inhibitor (GANT61) potently suppressed HCC PDO cell viability. In addition, there was a highly synergistic effect in vitro and in vivo on the suppression of cell viability and malignant properties when sorafenib and GANT61 were added to CD44-positive HCC PDOs and cell lines, respectively. Furthermore, the upregulation of CD44 and Hedgehog signaling induced by sorafenib was reversed by GANT61.

**Conclusions:**

GANT61 significantly suppressed Hedgehog signaling to reverse sorafenib resistance in CD44-positive HCC. The combination of sorafenib and Hedgehog signaling inhibitors might be effective in HCC patients with high CD44 levels as a personalized-medicine approach.

## Background

Hepatocellular carcinoma (HCC) is the most frequently diagnosed liver cancer and the sixth most common neoplasm worldwide [1, 2]. Sorafenib, a Food and Drug Administration (FDA)-approved first-line targeted agent, lengthens the median survival time from 7.9 months to 12.3 months [3, 4]. However, a majority of individuals show primary or secondary drug resistance. Because the mechanisms underlying resistance to sorafenib are rather unclear, it is vital to explore the causes of resistance and to implement a corresponding treatment strategy. Tumor-initiating cells (TICs), a minority subpopulation of cells that possess properties of progenitor cells, are present in numerous solid tumors [5, 6]. Studies based on human samples and in vitro models have demonstrated that TICs are highly aggressive and responsible for tumor metastasis, relapse and drug resistance, leading to disease-related mortality [7]. For example, Victoria Tovar and colleagues used transcriptomic analysis to uncover an increased number of TICs in tumors resistant to sorafenib [8], indicating that TICs might play an important role in sorafenib resistance. TICs are mainly identified through tumor formation in vivo, sphere formation in vitro, and specific cell markers. In HCC, CD44 is TIC marker most associated with the epithelial-mesenchymal transition (EMT), drug resistance and tumor formation in immunodeficient mice [9–11], suggesting that CD44 expression might be a crucial sign to guide clinical treatment. In addition, various stemness-related signals, such as Notch, Hippo, Wnt and Hedgehog signaling, are usually aberrantly activated in TICs, and drug resistance can be overcome by blocking these signals [12–14]. Among them, Hedgehog signaling, which is related to embryogenesis, participates in the differentiation of hepatocytes from endodermal progenitors [15]. It has been reported that activation of the Hedgehog pathway occurs in both the initiation and early progression stages of hepatocarcinogenesis and is associated with a poorly differentiated histopathology and relatively invasive phenotype [16, 17]. Thus, therapeutic strategies aimed at targeting TICs and stemness-related signals are particularly attractive, as they may hinder the development of drug resistance.

Nevertheless, another barrier in overcoming drug resistance is the lack of proper tumor models to evaluate the treatment efficacy of candidate agents. The currently commonly used cell line models of HCC cannot completely capture the features of primary tumors, such as cellular heterogeneity, cell-cell interactions and three-dimensional architecture. In contrast, recently developed patient-derived organoid (PDO) models, which have been described for liver [18], prostate [19], breast [20], and bladder [21] cancers, can overcome these limitations [22]. Moreover, these models conserve all parts of primary tumor cells, including TICs, and form functional organ-like structures [23, 24], providing an unparalleled strategy for studying tumor heterogeneity and creating an opportunity to tailor therapies for individual patients.

In this study, we established PDO models using samples from untreated HCC patients, and these HCC PDOs maintained the features of the original tumor, providing useful information for drug testing. Furthermore, we found that CD44-positive PDOs were significantly resistant to sorafenib. Blocking Hedgehog signaling significantly reduced cell viability and increased sensitivity to sorafenib, especially for CD44-positive HCC PDOs. Therefore, we propose that the combination of Hedgehog signaling inhibitors and sorafenib is a promising strategy for improving the curative effect in CD44-positive HCC patients.

## Methods

### Human HCC specimens

Tumor specimens were acquired from HCC patients who underwent surgical resection at the Department of Hepatobiliary Surgery, Peking University People’s Hospital, Beijing, China. All patients were first diagnosed with HCC and had not yet received therapy. The specimens were cut into two pieces of approximately 1 cm^3^: one random piece was fixed in formalin for histopathological analysis, and the other was processed into single cells for PDO culture.

### PDO culture

Briefly, each HCC specimen was minced on ice and digested with liberase (TM) (Roche) and 0.1% DNase I (Sigma) for 0.5 h–2 h at 37 °C on an orbital shaker. The incubation continued until the digestion preparation was visually inspected, after which the suspension was strained though a 75-μm nylon filter, and approximately 10 ml of Advanced DMEM/F12 (Gibco) with 10% fetal bovine serum (FBS) was added before centrifugation for 5 min at 300×g. The supernatant was removed, and the pellet was washed in precooled phosphate buffer saline (PBS), mixed with a growth factor-reduced (GFR) Matrigel matrix (Corning) and seeded in a 24-well or 96-well plate (Corning). After polymerization of the GFR Matrigel matrix, PDO culture medium (advanced DMEM/F12 supplemented with 1× GlutaMAX (Sigma), 10 mM HEPES (Sigma), 1× B-27 (Sigma), 1× N-2 (Sigma), 1.25 mM N-acetyl-l-cysteine (Sigma), 10 mM nicotinamide (Sigma), 10 nM recombinant human (Leu15)-gastrin I (Sigma), 50 ng/ml recombinant human EGF (Invitrogen), 100 ng/ml recombinant human FGF10 (PeproTech), 25 ng/ml recombinant human HGF (PeproTech), 10 μM forskolin (Tocris), 5 μM A8301 (Sigma), 10 μM Y27632 (Sigma) and 3 nM dexamethasone (Sigma)) was added [18, 25]. The culture medium was changed twice per week. The PDOs were passaged every 1–2 weeks after dissociation with Dispase (Corning). For storage, the PDOs were dissociated and resuspended in recovery cell culture freezing medium (Stem Cell) and frozen according to standard procedures.

### Cell lines and reagents

The HCC cell lines BEL7402, SNU423 and Hep3B were purchased from China Type Culture Collection (Shanghai, China) and cultured in RPMI-1640 medium (Sigma) supplemented with 10% FBS (Gibco), 100 U/ml penicillin and 100 mg/ml streptomycin. The following reagents were used: dibenzazepine (Selleck, S2711); Wnt-C59 (MCE, HY-15659); GANT61 (MCE, HY-13901); verteporfin (MCE, HY-B0146); sorafenib (Selleck, S1040) and purmorphamine (Selleck, S3042).

### Immunohistochemistry (IHC) and immunofluorescence (IF)

Tumor specimens or HCC PDOs were fixed in formalin or 4% paraformaldehyde, respectively, followed by dehydration and paraffin embedding. The sections were subjected to hematoxylin and eosin (H&E), IHC and IF staining. IHC was performed with Vectastain Elite ABC Kit (Vector Laboratories) following the manufacturer’s protocol. The Citrate buffer (pH 6.0) was used for antigen retrieval, and 0.3% NaHB_4_ was used for immunoperoxidase labeling. After washing with PBS three times, the sections were incubated with a primary antibody at 4 °C overnight. Incubation with the corresponding secondary antibody and a peroxidase-antiperoxidase complex was carried out for 1 h at room temperature. Immunoreactive sites were visualized with 3, 30-DAB. For IF, all procedures were carried out as performed for IHC, except that the cells were incubated with an IgG antibody conjugated with Alexa Fluor® 488 (1:1000) (Thermo Scientific) or an IgG antibody conjugated with Alexa Fluor® 555 (1:1000) (Thermo Scientific). The resulting signals were visualized using a confocal laser scanning microscope (Olympus BX61, Tokyo, Japan). The following primary antibodies were used: anti-AFP (Proteintech, 14,550–1-AP), anti-GPC3 (Abcam, ab129381), anti-EpCAM (Proteintech, 21,050–1-AP), anti-Ki-67 (CST, 9449), anti-CD44 (CST, 3570), anti-Sonic Hedgehog (Abcam, ab53281), anti-PTCH1 (Immunoway, YT3598), anti-Smoothened (Abcam, ab113438), and anti-GLI1 (Abcam, ab49314). To detect apoptosis, paraffin-embedded sections were deparaffinized and treated with DeadEnd Fluorometric TUNEL System (Promega) according to the manufacturer’s instructions.

### Western blotting

Protein lysates were obtained by homogenizing cells with RIPA (Merck with the Roche Complete protease inhibitor mixture). Proteins (20–40 μg) were separated by SDS-PAGE (10%) and transferred to a polyvinylidene difluoride (PVDF) membrane. After blocking with 5% skim milk, the membranes were incubated with a primary antibody at 4 °C overnight, followed by incubation with a horseradish peroxidase (HRP)-conjugated secondary antibody (CST,7074 and 7076) at room temperature for 1 h. Chemiluminescent HRP substrates (Millipore, Billerica, MA, USA) were used to visualize antibody binding. The following antibodies were used: anti-Sox2 (Abcam, ab92494), anti-c-Myc (CST, 5605), anti-Nanog (Abcam, ab109250), anti-Oct4 (Abcam, 181,557), anti-MEKK2 (Abcam, ab33918), anti-MEKK3 (Abcam, ab40756) and anti-GAPDH (Proteintech, 60,004–1-Ig).

### RNA isolation and quantitative real-time PCR (qPCR)

Total RNA was extracted from PDOs or HCC cells with TRIzol reagent (Invitrogen) following the manufacturer’s instructions. qPCR was performed to assess mRNA expression using the Bio-Rad CFX96 real-time PCR detection system.

### Drug treatment

HCC PDOs were seeded in 10 μl of GFR Matrigel matrix droplets in a 96-well plate (Corning) and cultured for 6 days. The PDOs were then treated with culture medium containing different drugs. Cell viability was detected using CellTiter-Glo 3D reagent (Promega,G9681). For HCC cell lines, cell viability was tested after treatment with purmorphamine, GANT61 or sorafenib, as indicated by alamarBlue™ (Invitrogen, DAL1025). The results were normalized to those for vehicle treatment (DMSO). All experiments were performed in technical (same screening run) and biological (different passages) duplicates and were subjected to stringent quality-control measures.

### Chou-Talalay analysis

Cell viability assays for the 20, 40, 60, 80 and 100% IC50 of each drug alone or in combination treatment were performed and tested with alamarBlue™. The data were input into the CompuSyn program (ComboSyn Inc., Paramus, NJ), as were the concentration ratios, to find the combination index for each combination to determine synergism or antagonism. All data were collected and analyzed as directed by the Chou-Talalay method [26]. CI is a quantitative measure of the degree of drug interaction, whereby CI < 1 indicates synergism, CI > 1 indicates antagonism, and CI = 1 indicates an additive effect.

### Colony formation assay and cell invasion assay

For colony formation, HCC cell were plated in regular medium in 6-well plates at 37 °C with 5% CO_2_. After 1–2 weeks of incubation, the cells were fixed with 4% paraformaldehyde, stained with 0.1% crystal violet, and washed with PBS before analysis. The relative numbers of colonies were counted under a microscope. A cell invasion assay was performed using 8-μm pore-size hanging cell inserts (Corning). First, the bottom of the upper chamber was coated with Matrigel matrix (BD Biosciences) (10 mg/ml) according to the manufacturer’s protocol, and 1 × 10^5^ cells in serum-free DMEM were seeded in the upper chamber; the lower chamber was filled with 20% FBS. After a 48 h incubation, the noninvaded cells on the upper surface of the filter were removed with a cotton swab, and the invasive cells were fixed with 4% paraformaldehyde and stained with 0.1% crystal violet. The number of invasive cells on the lower surface of the membrane was counted under a microscope.

### Sphere formation assay

HCC cells were seeded in an Ultralow-attachment 6-well plate (Corning) in spheroid formation medium (advanced DMEM/F12 supplemented with 1× N2, 1× B27, 20 ng/ml hEGF, and 20 ng/ml bFGF (PeproTech)). After 7 days of incubation, spheres were evaluated by microscopy and extracted for protein analysis.

### Animals model

For the xenograft experiment, 1 × 10^6^ BEL7402 or Hep3B cells were injected subcutaneously into to 5 week-old BALB/c nu/nu mice. Tumor growth was monitored twice a week by measuring the length and width of the tumor. The tumor volume was calculated according to the formula V (cm^3^) = 1/2 × Length × Width^2^. For drug treatment, tumors were allowed to reach approximately 60 mm^3^ in size before drug treatment. The mice were randomized into 4 groups: control (vehicle), sorafenib (30 mg/kg/day, administered orally), GANT61 (40 mg/kg, twice a week, administered intraperitoneally), and sorafenib+GANT61 treatment. After approximately 4 weeks, the mice were killed under anesthesia. The tumors were collected and fixed in 10% formalin and embedded in paraffin. H&E staining and IF were performed on sections from embedded samples.

### Statistical analysis

Data are shown as the mean ± SD Statistical evaluations between two groups were performed by Student’s t-test. Experiments with more than three groups were evaluated by one-way ANOVA followed by Bonferroni’s test. Overall survival curves were plotted by the Kaplan-Meier method and compared using the log-rank test. A *P* value less than 0.05 was considered statistically significant.

## Results

### Establishment of HCC PDOs in vitro

**Fig. 1:**
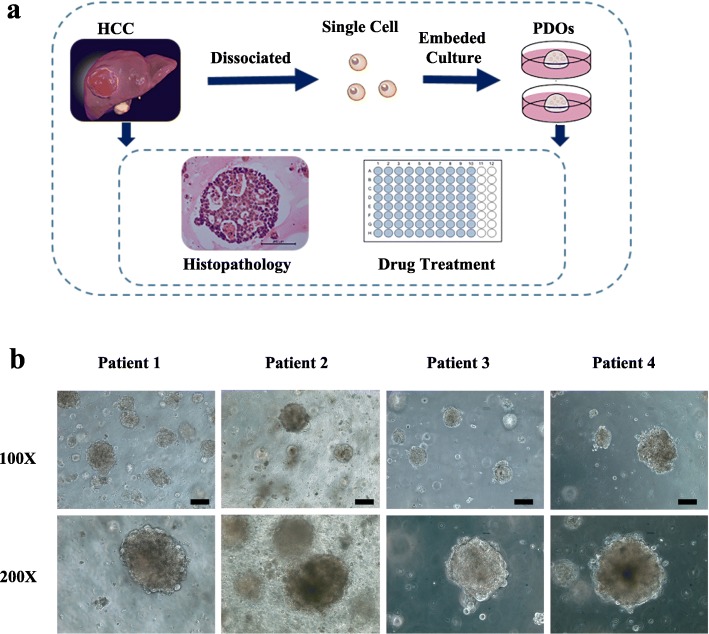
Establishment of HCC PDOs *in vitro.* **a** Workflow schematic. HCC specimens were obtained from patients who underwent surgery and processed as described in the Methods section to establish HCC PDOs. **b** Representative bright-field images of HCC PDOs from four individuals imaged with different magnification factors. Scale bar, 200 μm

**Fig. 2:**
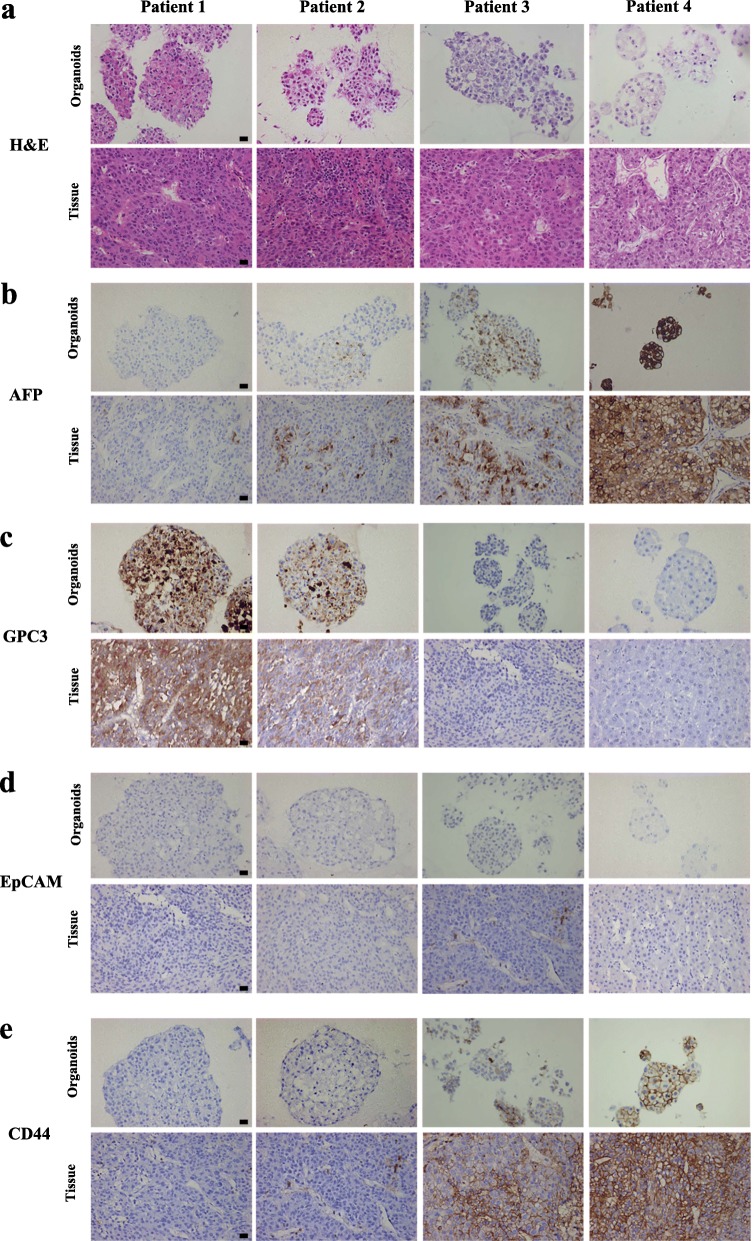
Histopathological characteristics of HCC PDOs and the original tumors. **a** H&E-stained histological sections of HCC PDOs and the corresponding tumors. The cell morphology and arrangement of the original tumors were maintained in the corresponding HCC PDOs. Scale bar, 50 μm. **b** AFP expression detected by immunohistochemistry in HCC PDOs and original tumors. Scale bar, 50 μm. **c** GPC3 expression detected by immunohistochemistry in HCC PDOs and original tumors. Scale bar, 50 μm. **d** EpCAM expression detected by immunohistochemistry in HCC PDOs and original tumors. Scale bar, 50 μm. **e** Expression of the TIC marker CD44 detected by immunohistochemistry in HCC PDOs and original tumors. Scale bar: 50 μm

First, we collected HCC specimens from newly diagnosed patients. The specimens were split into two parts that were processed for histological diagnosis or PDO derivation, allowing comprehensive characterization of the samples. The workflow is shown in Fig. 1a. After culture in vitro for 1 to 2 weeks, the structures of the PDOs were visually observed. Regarding morphology, the HCC PDOs were dense spheres composed of several cells to hundreds of cells that sometimes developed a gland-like structure, as shown in Fig. 1b. The background diseases of the individual patients included the most common risk factors for HCC, such as viral hepatitis and alcoholic liver disease (ALD) (Table 1). Next, histological analysis of paraffin-embedded sections was performed to explore whether the HCC PDOs preserved the histological features of the original tumors and the results showed that the phenotypic features of the PDOs resembled those of the corresponding tumors (Fig. 2a). We next assessed the expression of alpha-fetoprotein (AFP), a well-established marker of HCC, and the cellular location and intensity of AFP in the PDOs and original tumors were consistent (Fig. 2b). The additional biomarker Glypican 3 (GPC3), which is widely used for the diagnosis of HCC, exhibited the same expression pattern in the PDOs and tumors (Fig. 2c), and the biliary marker EpCAM was absent from both (Fig. 2d). In addition, the TIC marker CD44 was tested and its expression was maintained in the PDOs (Fig. 2e). Hence, we conclude that the HCC PDOs retained the histological features and expression profiles of the tumors from which they were derived.

**Table 1:** Patients’ information

Patients Data						
Patient Sample	Gender	Age	Liver Disease(s)	Cirrhosis	Histological Grade	AFP(ng/ml)
HCC-1	F	54	HBV	Yes	Mod/Well Differentiated	5.86
HCC-2	M	61	HCV ALD	Yes	Mod Differentiated	21.63
HCC-3	M	73	ALD	Yes	Mod Differentiated	1210.00
HCC-4	M	58	HBV	yes	Poorly Differentiated	6524.00

Histological grade were determined by H&E-stained sections by experienced hepato-pathologists. Serum alpha-fetoprotein (AFP) concentrations were obtained from the clinical charts of the patients. *AFP* alpha-fetoprotein, *ALD* alcoholic liver disease, *HBV* hepatitis B virus, *HCC* hepatocellular carcinoma, *HCV* hepatitis C virus

### HCC PDOs with different CD44 levels exhibited different sensitivities to sorafenib

**Fig. 3:**
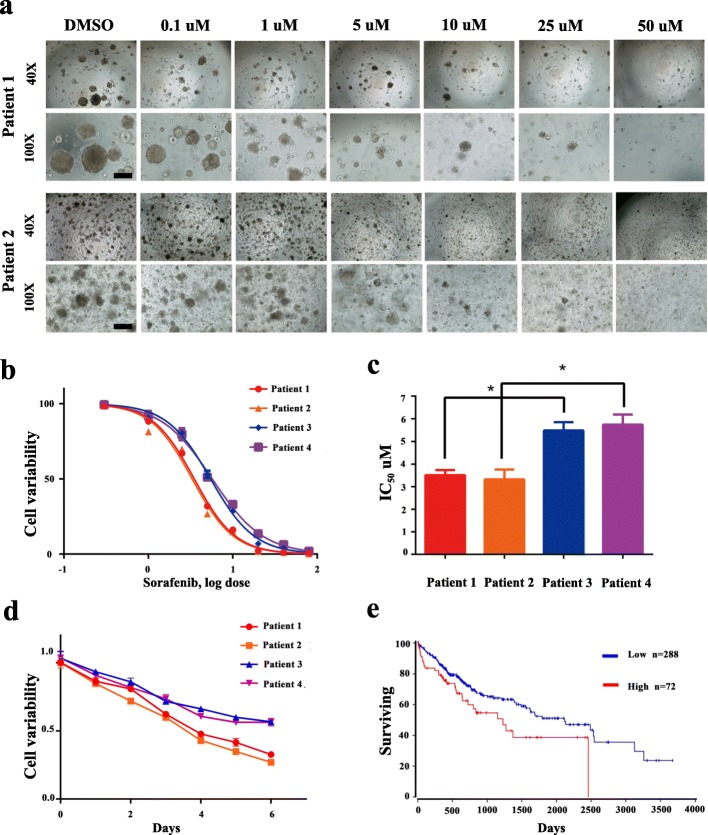
HCC PDOs with different CD44 levels exhibited different sensitivitities to sorafenib. **a** Representative bright-field images of HCC PDOs (patient 1 and patient 4) that were exposed to sorafenib at the indicated concentration for 6 days are shown. DMSO-treated PDOs were used as controls. Scale bar, 200 μm. **b** Sorafenib reduced the viability of 4 HCC PDOs in a dose-dependent manner. DMSO-treated PDOs were used as controls, and the mean of two independent experiments performed with three duplicates is shown. **c** The differential IC50 values (μM) of 4 HCC PDOs are shown as the mean ± SD. **d** HCC PDOs were exposed to sorafenib for 6 days, and cell viability was recorded daily. **e** Kaplan-Meier overall survival curves for patients with low or high CD44 expression from TCGA are shown. (* p < 0.05, P < 0.05 is considered statistically significant)

We continued to evaluate whether PDOs are an appropriate model for drug sensitivity testing. HCC PDOs were treated with a dilution series of sorafenib for 6 days, and cell viability was evaluated with CellTiter-Glo reagent. As represented by bright-field images and half-maximal inhibitory concentration (IC50) values, sorafenib inhibited HCC PDO growth in a dose-dependent manner, with IC50 values that varied from 3.31 to 5.73 μM (Fig. 3a, b and c). In addition, CD44-positive PDOs (patients 3 and 4) were obviously resistant to sorafenib, with higher IC50 values than those of CD44-negative PDOs (patients 1 and 2) (Fig. 3b and c). Similarly, when sorafenib treatment was applied for different periods of time, the decrease in cell viability induced by sorafenib was delayed in the CD44-positive PDOs (Fig. 3d). According to The Cancer Genome Atlas (TCGA) database, CD44-positive HCC patients have a poorer prognosis than do CD44-negative HCC patients (Fig. 3e). These data show that HCC PDOs constitute potential in vitro models that may be used to detect patient-specific sensitivities to drugs and that CD44-positive HCC PDOs are prone to resistance to sorafenib treatment.

### Effects of TIC-related signaling inhibitors on the cell viability of HCC PDOs

**Fig. 4:**
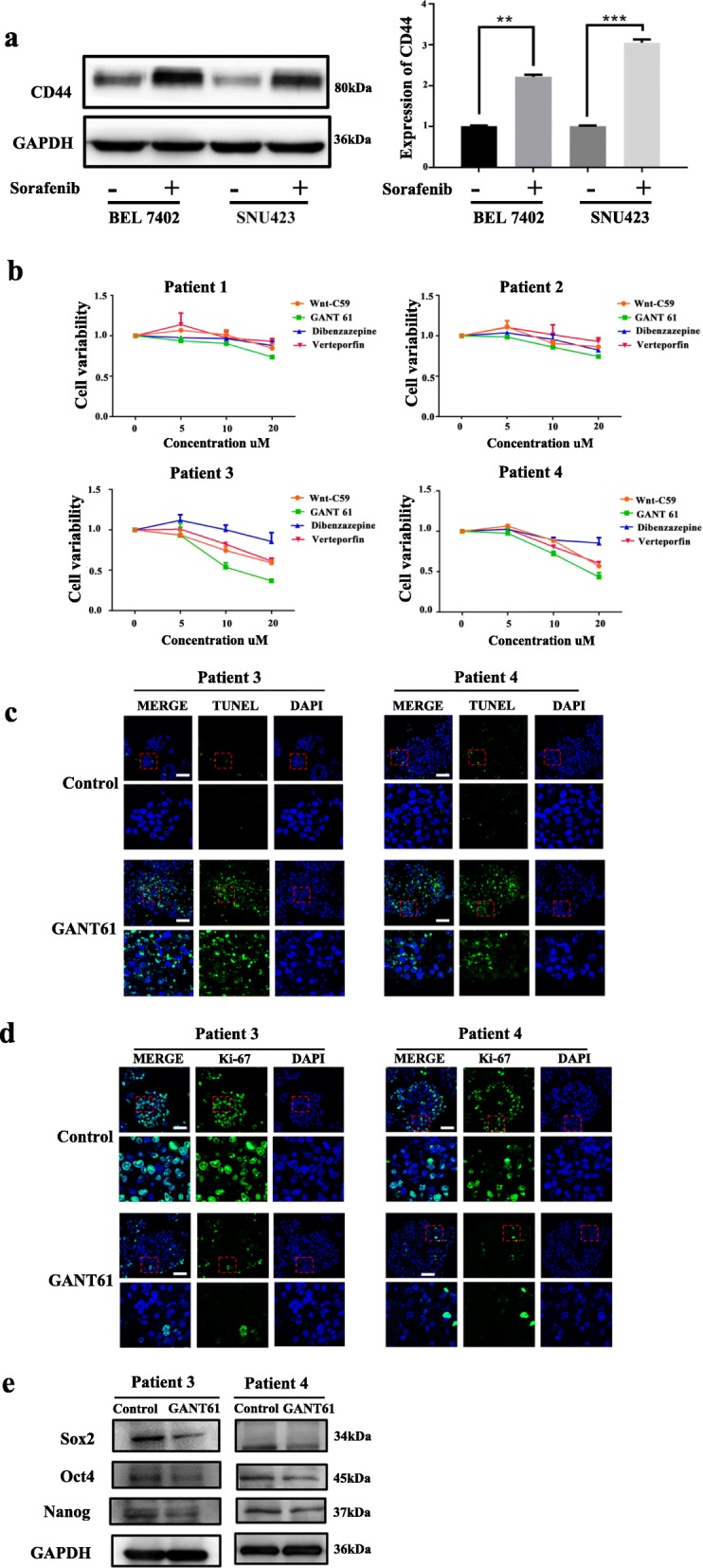
Effects of TIC-related signaling inhibitors on the cell viability of HCC PDOs. **a** Sorafenib treatment affected the CD44 levels at the protein or RNA levels in HCC cell lines; **b** 4 HCC PDOs were treated with a Notch signaling inhibitor (YO-01027), Wnt signaling inhibitor (Wnt-C59), Hippo signaling inhibitor (verteporfin), or Hedgehog signaling inhibitor (GANT61) for 6 days and cell viability was recorded. **c** Immunofluorescence images of HCC PDOs after treatment with DMSO or GANT61 for 6 days. The cells were stained for TUNEL (green) and stained with DAPI (blue). Scale bar: 50 μm. **d** Immunofluorescence images of HCC PDOs after treatment with DMSO or GANT61 for 6 days. The cells were stained for Ki-67 (green) and stained with DAPI (blue). Scale bar: 50 μm. **e** Protein expression of Oct4, Sox2 and Nanog in HCC PDOs after treatment with GANT61 (10 μM) determined by Western blotting. (** p < 0.01, *** p < 0.001, P < 0.05 is considered statistically significant)

Furthermore, we observed upregulation of CD44 at both the protein and RNA levels after sorafenib treatment in HCC cell lines (BEL 7402 and SNU423) (Fig. 4a). Considering that CD44 is an important TIC marker involved in various stemness signaling pathways, we compared the efficacy of various stemness signaling inhibitors on CD44-positive or CD44-negative HCC PDOs. Treatment with a Notch signaling inhibitor (dibenzazepine), Wnt signaling inhibitor (Wnt-C59), or Hippo signaling inhibitor (verteporfin) produced marginal effects on cell viability among all HCC PDOs. The Hedgehog signaling inhibitor (GANT61) obviously decreased cell viability in a dose-dependent manner (Fig. 4b). In addition, the inhibitory effect of GANT61 in CD44-positive PDOs was stronger than that in CD44-negative PDOs. GANT61 treatment also notably decreased cell proliferation and increased apoptosis in HCC PDOs (Fig. 4c and d). The levels of TIC-related proteins, such as Sox2, Nanog and Oct4, also decreased after GANT61 treatment (Fig. 4e). The above results suggest that GANT61 effectively suppresses the stemness of HCC PDOs by blocking Hedgehog signaling.

### A hedgehog signaling inhibitor enhanced CD44-positive HCC PDO sensitivity to sorafenib

**Fig. 5:**
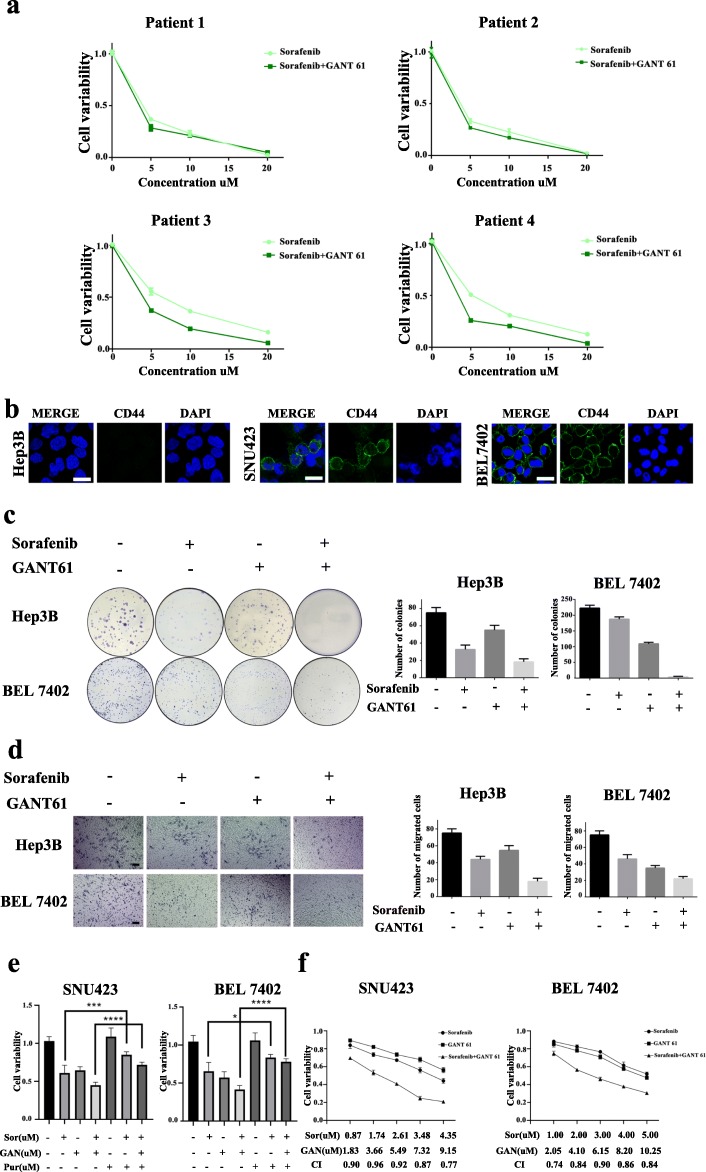
Hedgehog signaling inhibitor promoted sensitivity to sorafenib in CD44-positive HCC PDOs. **a** 4 HCC PDOs were treated with sorafenib in the presence or absence of GANT61 for 6 days. Cell viability was determined using CellTiter-Glo reagents. **b** IF images of CD44 in HCC cell lines (Hep3B, SNU423 and BEL7402). Scale bar: 25 μm. **c** Representative bright-field images of 2 HCC cell lines (Hep3B and BEL7402) treated with DMSO, sorafenib (10 μM), GANT61 (5 μM) or a combination of sorafenib and GANT61 for 6 days are shown. After fixation with 100% ethanol, the colonies were stained with crystal violet. The number of colonies was counted. **d** Representative bright-field images of 2 HCC cell lines (Hep3B and 7402) treated with DMSO, sorafenib (10 μM), GANT61 (5 μM) or a combination of sorafenib and GANT61 for 6 days are shown. After fixation with 100% ethanol, the invasive cells were stained with crystal violet. The number of migrated cells was counted. Scale bar: 500 μm. **e** The viability of SNU423 and BEL7402 HCC cells was tested and treated with sorafenib, purmorphamine, GANT61 or the combination as indicated for 48 h. **f** The viability of SNU423 and BEL7402 HCC cells was tested, after treatment with sorafenib, GANT61 or a combination of sorafenib and GANT61. The Chou-Talalay combination index (CI) was calculated. (*p < 0.05, *** p < 0.001, **** p < 0.0001, P < 0.05 is considered statistically significant)

To assess whether a synergistic effect mediated by cotreatment of Hedgehog signaling inhibitors and sorafenib affect HCC PDO cell viability, we cultured HCC PDOs with sorafenib in the absence or presence of Hedgehog signaling inhibitors. After treatment with sorafenib alone, the viability of CD44-negative PDOs decreased significantly compared to CD44-positive PDOs. Although the combination of sorafenib and GANT61 had little additive effect on CD44-negative HCC PDOs, a highly synergistic effect was observed after cotreatment of sorafenib and GANT61 in CD44-positive HCC PDOs (Fig. 5a). Next, we confirmed our conclusion in HCC cell lines with different CD44 levels (Fig. 5b) using colony formation and cell invasion assays. As shown in Fig. 5c, the combination of sorafenib and GANT61 greatly decreased colony size and number, especially in the CD44-positive BEL 7402 cells. Similarly, we examined the property of invasiveness and found that the estimated number of viable BEL7402 cells decreased significantly after treatment with sorafenib in the presence of GANT61 (Fig. 5d). Next, a rescue experiment was designed to assess the impact of Hedgehog signaling activation on sorafenib resistance, HCC cells were treated with sorafenib in the presence or absence of GANT61 or purmorphamine (Hedgehog signaling agonist). As shown in Fig. 5e, cell viability was significantly higher in the presence of purmorphamine, suggesting that activation of Hedgehog signaling significantly decreased the inhibitory effects of the combination of sorafenib and GANT61 in CD44-positive HCC cell lines. To further investigate drug interaction types between sorafenib and GANT61, we evaluated combination index (CI) values using CompuSyn software. As shown in Fig. 5f, the CI values of the combination treatment of sorafenib and GANT61 in different CD44-positive HCC cell lines were smaller than 1, indicating that sorafenib and GANT61 act synergistically in HCC cells. Thus, there is a synergistic effect between sorafenib and GANT61, and GANT61 increases sensitivity to sorafenib in CD44-positive HCC cells by decreasing cell viability and inhibiting malignant transformation.

### Combined treatment with sorafenib and GANT61 significantly reduces HCC tumorigenesis in vivo

**Fig. 6:**
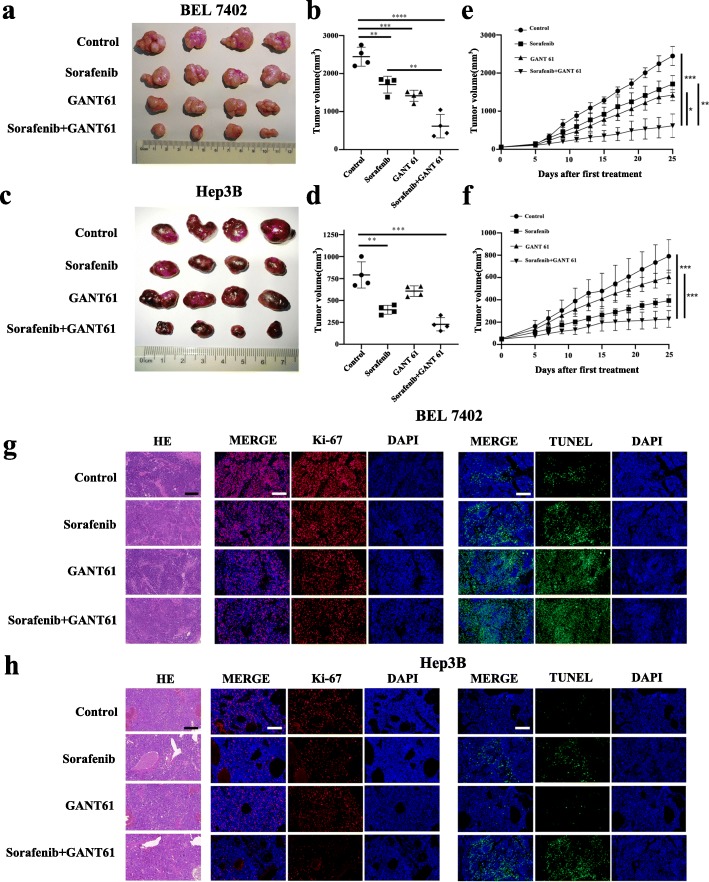
Combined treatment with sorafenib and GANT61 significantly reduces HCC tumorigenesis *in vivo.* **a** (**c**) Photographs illustrating tumors in xenografts of BEL7402 or Hep3B cell lines under different treatments. **b** (**d**) The end tumor volume of BEL7402 or Hep3B cell lines under different treatment. **e** (**f**) Tumor growth of BEL7402 or Hep3B cell lines was monitored and shown under different treatments. **g** (**h**) Representative images showing H&E and immunofluorescence staining of Ki67 and TUNEL on tumors of different treatment are shown. Scale bar, 50 μm. (*p < 0.05, **p < 0.01, *** p < 0.001, **** p < 0.0001, P < 0.05 is considered statistically significant)

Animals subjected to subcutaneous injection of Bel 7402 or Hep3B cells were randomly divided into four treatment groups: 1. control, 2. sorafenib, 3. GANT61 and 4. sorafenib and GANT61 combination. For CD44-positive BEL 7402 cells, the results showed a significant reduction in tumor volume compared to the sorafenib in the combination treatment groups (Fig. 6a and b). For CD44-negative Hep3B cells, a reduction in tumor volume was not obvious with GANT61 administration but obvious in the combination group (Fig. 6c and d). The results also showed that the combination of sorafenib and GANT61 exerted the most potent effect in inhibiting tumor formation, especially for CD44-positive HCC cells (Fig. 6e and f). Consistently, tumor volume and dimensions were significantly reduced in the combination treatment group. IHC analysis revealed that Ki-67 positive cells were decreased and that apoptotic cells were increased in tumors treated with combination treatment (Fig. 6g and h). Thus, there is also a synergistic effect between sorafenib and GANT61to reduce HCC tumorigenesis in vivo.

### GANT61 reversed the increase in hedgehog signaling proteins and CD44 caused by sorafenib treatment

**Fig. 7:**
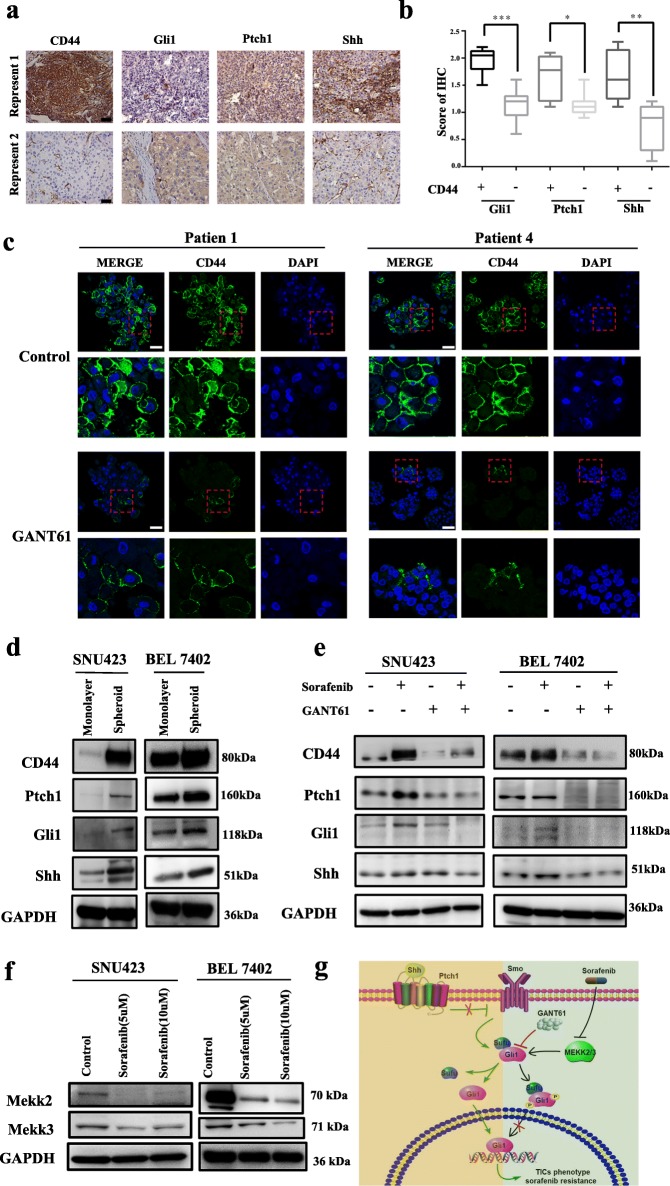
GANT61 reversed the increase in Hedgehog signaling proteins and CD44 caused by sorafenib treatment. **a** Representative bright-field images of immunohistochemical staining for CD44, Gli1, Ptch1 and Shh in patient tumor samples showing low or high expression. Scale bar: 50 μm. **b** The result of immunohistochemical staining in a box diagram. **c** Immunofluorescence images of HCC PDOs after treatment with DMSO or GANT61 for 6 days. The cells were stained for CD44 (green) and stained with DAPI (blue). **d** The levels of CD44 and Hedgehog-related proteins in cell lines (SNU423 and BEL7402) grown as monolayer cells or spheroids. **e** Western blot analysis showed the levels of CD44 and Hedgehog-related proteins in cell lines (SNU423 and 7402) after treatment with DMSO (control), sorafenib, GANT61 or a combination of sorafenib and GANT61. **f** Western blot analysis showed the expression of MEKK2 and MEKK3 after sorafenib treatment at different concentrations in cell lines (SNU423 and BEL7402). **g** Illustration of the GANT inhibits Gli1to reverse the resistance of sorafenib in HCC. (*p < 0.05, ** p < 0.01, *** p < 0.001, P < 0.05 is considered statistically significant)

To examine the relationship between CD44 levels and Hedgehog signaling activation, 20 patient specimens were evaluated by IHC. The results showed that overexpression of CD44 was frequently accompanied by high levels of Patched 1 (Ptch1), Smoothened (Smo), and Glioma-associated oncogene homolog-1 (Gli1) (Fig. 7a and b). In addition, levels of CD44 were obviously decreased after Hedgehog signaling inhibition in CD44-positive PDOs (Fig. 7c). Both of these results suggest that CD44 levels correlate positively with Hedgehog signaling activation. We then examined levels of CD44 and Hedgehog signaling proteins (Ptch1, Shh and Gli1) in HCC cell lines grown in a monolayer or in a spheroid condition by Western blot analysis and found that levels of CD44 and Hedgehog signaling proteins were higher in the spheroid cells than in the monolayer cells (Fig. 7d). Furthermore, HCC cell lines were treated with DMSO (control), sorafenib, GANT61, or sorafenib and GANT61 combination for 24 h, and expression of Ptch1, Gli1, Shh and CD44 was examined. As shown in Fig. 6d, when sorafenib was added to the cell lines, levels of Ptch1, Gli1, Shh and CD44 were upregulated significantly, suggesting that stimulation with sorafenib promotes conversion into a stem/progenitor cell phenotype by activating Hedgehog signaling, which might be involved in sorafenib resistance. In contrast, GANT61 treatment markedly suppressed Hedgehog signaling and downregulated CD44 levels. In addition, cotreatment of sorafenib and GANT61 prevented activation of Hedgehog signaling and overexpression of CD44 induced by sorafenib administration (Fig. 7e). Regardless, the mechanism by which sorafenib activates Hedgehog signaling has not yet been clarified. In this study, we found that sorafenib is able to inhibit expression of MEKK2/3 (Fig. 7f). Furthermore, it is reported that MEKK2/3 promotes Gli1 cytoplasmic retention, resulting in Hedgehog signaling inhibition [27]. Therefore, we infer that sorafenib activate Hedgehog signaling through inhibition of MEKK2/3, though the underlying mechanisms by which sorafenib affects MEKK2/3 require further research (Fig. 7g). These data suggest that CD44-positivity is frequently accompanied by Hedgehog signaling activation and that GANT61 obviously restrains the upregulation of Hedgehog signaling proteins resulting from sorafenib treatment.

## Discussion

This study for the first time explored a crucial role of Hedgehog signaling in sorafenib resistance in a subpopulation of CD44-positive HCC. We established four HCC PDOs from different patients, demonstrating that these in vitro models completely maintain the features of the original tumors and respond to drug treatment. Furthermore, CD44-positive HCC PDOs were more prone to sorafenib resistance. Compared with other classic TIC-related inhibitors, the inhibitor of Hedgehog signaling obviously decreased cell viability and increased apoptosis in HCC PDOs. In addition, cotreatment with sorafenib and Hedgehog signaling inhibitors had a dramatic synergistic effect of inhibiting the proliferation of HCC cells, especially CD44-positive cells, both in vitro and in vivo. Moreover, properties of colony formation and invasiveness were attenuated following Hedgehog signaling blockade. Finally, high levels of CD44 were relatively frequently accompanied by Hedgehog signaling activation. Sorafenib treatment increased CD44 levels, which was reversed by Hedgehog signaling inhibition. The above results suggest that the sensitivity of HCC PDOs to sorafenib can be indicated by CD44 levels, which are related to the activation of Hedgehog signaling. Thus, Hedgehog signaling is promising as a combinational therapeutic strategy for patients with high CD44 levels.

HCC exhibits high molecular heterogeneity, owing to the coexistence of different subsets with different sensitivities to targeted therapies, and accurate individualized treatment is urgently needed [28]. Nonetheless, preclinical tumor research tools show substantial limitations. Several cell lines have been used as normal in vitro models to represent different tumors, but they fail to reflect the heterogeneous tumor context and predict the clinical outcomes of different individuals [29]. Another classic tumor model is the patient-derived xenograft (PDX) model, which preserves tumor heterogeneity to some extent but is labor and time intensive, cannot be expanded and exhibits inefficient generation [30]. The appearance of PDOs overcomes many of these limitations [22]. PDO models consist of different subsets within tumors and maintain the histological features and expression profiles of the tumors from which they were derived. In this study, the success rate of PDOs was approximately 50%, higher than that reported in a study of biopsied HCC specimens (33%) [25], possibly due to the small sample size and hepatocyte contamination. All HCC PDOs were generated from moderate or poorly differentiated tumors, which was in line with previous studies [18], suggesting that cell viability is required for the generation of PDOs. In addition, expression of the TIC marker CD44 persists in HCC PDOs, providing an opportunity to study the differentiation of drug efficacy in primary HCC cells with different CD44 levels.

Resistance to targeted drugs, such as sorafenib, in HCC patients is a major problem. Over the last decade, acknowledged mechanisms of resistance to targeted drugs have included continuous activation of target genes owing to secondary mutations [31], enhanced gene expression [32] or abnormal regulation of compensatory signaling, such as aberrant activation of PI3K/AKT by MEK or mTOR inhibition [33, 34]. Another resistance mechanism, gain of stem cell phenotypic features, within the heterogeneous tumor cell subgroup is receiving increasing attention [7]. CD44 is acknowledged as a vital TIC marker and is associated with a poor survival rate in numerous types of tumors [35]. Bera and colleagues demonstrated that gemcitabine treatment induces pancreatic cancer cell lines to undergo an EMT process and convert from CD44 negativity to CD44 positivity [36]. Similarly, we found that the level of CD44 can indicate the sensitivity of HCC PDOs to sorafenib. Furthermore, a switch in the expression levels of CD44 may be observed both in HCC PDO and in cell lines after sorafenib treatment. Thus, sorafenib treatment might induce conversion to a CD44-positive phenotype, including the acquisition of TIC features and increased insensitivity to drugs. On the other hand, our results showed that GANT61 treatment significantly decreased levels of CD44 in HCC PDOs and cotreatment of sorafenib and that GANT61 inhibited the tumor growth both in vitro and in vivo. Therefore, suppression or reversion of this CD44 profile conversion might be a crucial strategy for enhancing treatment efficacy.

In addition to ITC markers, stemness-related signals, including Wnt, Hippo, Notch and Hedgehog pathways, also play vital roles in the acquisition of ITC phenotypes. In this study, we found that inhibition of Hedgehog signaling maximally reduced the proliferation of HCC PDOs. Activation of Hedgehog signaling is mainly caused by binding of the Hedgehog ligand to the receptor Ptch1, which in turn relieves inhibition of the Smoothened (Smo). Smo then activates transcription of Gli1, which enters the nucleus to promote expression of target genes [37]. HBV infection, a well-known risk factor for HCC, has been reported to regulate posttranslational activation of Hedgehog signaling, which leads to hepatocarcinogenesis [38]. It has been confirmed that cell proliferation and self-renewal can be inhibited and that drug sensitivity to temozolomide can be increased by blocking Hedgehog signaling in glioma [39]. Although Hedgehog signaling activation has been demonstrated in HCC [40, 41] and has been shown to be a key regulator of autophagy in HCC cells [42], there is little data on the exact roles of Hedgehog signaling in HCC TIC conversion and sorafenib resistance. In this study we first demonstrated that Hedgehog activation may occur in a subpopulation of HCC patients who are CD44 positive and that blocking this activation can significantly increase sorafenib sensitivity. By activating Hedgehog signaling, purmorphamine reversed the inhibitory of cotreatment of sorafenib and GANT61 in HCC. Gli1 is a pivotal component of Hedgehog signaling; its expression is precisely regulated in the process of cell proliferation, and its deregulation leads to tumorigenesis. Gli1 typically binds to with Sufu to form a complex; when Hedgehog signaling is activated, the Sufu-Gli1 complex dissociates, and Gli1 is activated [43]. Recently, it was reported that silencing MEKK2/3 promotes expression of Hedgehog signaling-related proteins [44]. Further research has found that MEKK2/3 enhances the Sufu-Gli1 interaction, resulting in Gli1 cytoplasmic retention, which occurs mechanistically via Gli1 phosphorylation by MEKK2/3 at multiple Ser/Thr sites [27]. Interestingly, our results indicate that sorafenib obviously decreases levels of MEKK2/3 in cell lines in a dose-dependent manner. Combined with the ability of sorafenib to induce activation of Hedgehog signaling, this observation indicates that sorafenib might suppress MEKK2/3 to promote Hedgehog signaling activation, resulting in the upregulation of CD44, acquisition of a progenitor cell phenotype and sorafenib resistance in HCC cells. Therefore, we hypothesize that sorafenib suppresses MEKK2/3, after which Gli1, which is not phosphorylated by MEKK2/3, enters the nucleus and promotes expression of stemness-related genes, converting the cell to the stem/progenitor cell phenotype and resulting in resistance to sorafenib (Fig. 6g).

## Conclusion

In conclusion, we defined a subpopulation of HCC with CD44-positive cells that exhibited sorafenib resistance. Hedgehog signaling inhibition reversed sorafenib resistance in CD44 positive PDOs. Therefore, we consider Hedgehog signaling inhibitors to be ‘defensive treatments’ that should be used with sorafenib in CD44-positive patients, not only to improve the drug sensitivity of HCC, but also to prevent acquired drug resistance.

## Data Availability

The datasets generated/ analysed during the current study are available.

## Abbreviations

AFP: Alpha-fetoprotein
ALD: Alcoholic liver disease
HBV: Hepatitis B virus
HCC: Hepatocellular carcinoma
HCV: Hepatitis C virus
PDOs: Patient-derived organoids
TCGA: The Cancer Genome Atlas
TICs: Tumor initiating cells
